# Molecular Dynamics Simulation Study of Conformational Changes of Transcription Factor TFIIS during RNA Polymerase II Transcriptional Arrest and Reactivation

**DOI:** 10.1371/journal.pone.0097975

**Published:** 2014-05-19

**Authors:** Changsun Eun, Juan Manuel Ortiz-Sánchez, Lintai Da, Dong Wang, J. Andrew McCammon

**Affiliations:** 1 Howard Hughes Medical Institute, University of California San Diego, La Jolla, California, United States of America; 2 Institute of Physical and Theoretical Chemistry, Goethe University Frankfurt, Frankfurt, Germany; 3 Skaggs School of Pharmacy and Pharmaceutical Sciences, University of California San Diego, La Jolla, California, United States of America; 4 Department of Chemistry, The Hong Kong University of Science and Technology, Clear Water Bay, Kowloon, Hong Kong; 5 Department of Chemistry and Biochemistry, University of California San Diego, La Jolla, California, United States of America; 6 Department of Pharmacology, University of California San Diego, La Jolla, California, United States of America; University of Akron, United States of America

## Abstract

Transcription factor IIS (TFIIS) is a protein known for catalyzing the cleavage reaction of the 3′-end of backtracked RNA transcript, allowing RNA polymerase II (Pol II) to reactivate the transcription process from the arrested state. Recent structural studies have provided a molecular basis of protein-protein interaction between TFIIS and Pol II. However, the detailed dynamic conformational changes of TFIIS upon binding to Pol II and the related thermodynamic information are largely unknown. Here we use computational approaches to investigate the conformational space of TFIIS in the Pol II-bound and Pol II-free (unbound) states. Our results reveal two distinct conformations of TFIIS: the closed and the open forms. The closed form is dominant in the Pol II-free (unbound) state of TFIIS, whereas the open form is favorable in the Pol II-bound state. Furthermore, we discuss the free energy difference involved in the conformational changes between the two forms in the presence or absence of Pol II. Additionally, our analysis indicates that hydrophobic interactions and the protein-protein interactions between TFIIS and Pol II are crucial for inducing the conformational changes of TFIIS. Our results provide novel insights into the functional interplay between Pol II and TFIIS as well as mechanism of reactivation of Pol II transcription by TFIIS.

## Introduction

**Figure 1:**
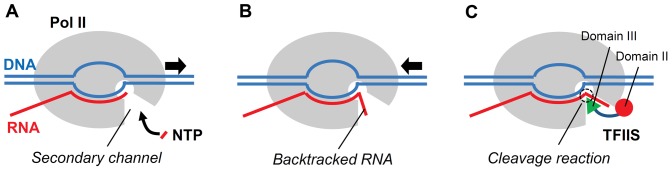
Schematic diagrams of the Pol II Elongation Complex (EC). (A) Transcribing state. (B) Backtracking state. (C) Reactivation intermediate state. The arrows in (A) and (B) indicate the moving direction of the Pol II relative to the RNA. Here, NTP stands for nucleoside triphosphate, a building block of RNA.

RNA polymerase II (Pol II) is a key enzyme responsible for transcribing messenger RNA (mRNA) from the DNA template in eukaryotic cells (see Figure 1A). During the transcription elongation process, upon each round of nucleotide addition, Pol II translocates forward along the template DNA strand by one base pair (bp) step (from the pre-translocation state to the post-translocation state) in order to make its active site available for the next round of nucleotide addition [1]. The forward translocation process can be interrupted by several factors such as template DNA damage and transcript misincorporation [2]. These factors can induce the backtracked state of Pol II [3], [4], which can potentially lead to transcriptional pausing and arrest.

Notably, the backtracked state of Pol II is necessary for proofreading the newly synthesized RNA transcript [5], [6]. In the case of misincorporation, it is thermodynamically favorable for Pol II to move backward and to allow its RNA transcript to enter the secondary channel as illustrated in Figure 1B. To resume the transcription process, the backtracked RNA 3′-end nucleotides are removed from the nascent RNA chain, which is achieved through the intrinsic cleavage reaction or through the TFIIS-catalyzed cleavage reaction (see Figure 1C). The cleavage reaction catalyzed by TFIIS is much faster than the intrinsic cleavage reaction and is a dominant process when more than one RNA 3′-end nucleotides are backtracked [4].

Experimental studies have provided molecular details of structure and biological function of TFIIS. TFIIS is composed of three major domains, termed I, II, and III. Early NMR studies revealed the structures of yeast TFIIS (yTFIIS) domain I [7] and domain II [8], and human TFIIS (hTFIIS) domain III [9] individually in the absence of Pol II. In addition to this structural information, some biological functions of these domains have also been reported. Specifically, it was experimentally suggested that a highly conserved motif (QTRSADE) in the domain III directly participates in the catalytic reaction [10], while the domain I was found to be dispensable for the catalytic function of TFIIS on transcript cleavage [11]. In addition, mutagenesis analyses revealed that substitutions of the residues Asp (D) and Glu (E) in this motif with other residues can dramatically reduce the cleavage activities of TFIIS [10]. With such mutants, the recent X-ray crystal structures of the Pol II-TFIIS complex clearly showed that the tip of the domain III is inserted into the active site of Pol II through the secondary channel [4], [12]. Furthermore, the crystal structures indicate that the domain II interacts with the Pol II Rpb1 domain through one of its three helix bundles, as is also suggested by previous mutagenesis results [11]. More interestingly, the domains II and III are connected by a long alpha helix. It is noteworthy that this linker region has been suggested to be largely unfolded and flexible in the unbound state of TFIIS since no obvious secondary structure was detected in previous NMR studies [11]. This indicates that TFIIS experiences a significant conformational change upon binding with Pol II.

Although experiments have provided insights into the structural and functional features of TFIIS, several important issues have not yet been addressed: what are the molecular dynamics of Pol II-TFIIS complex and thermodynamics associated with TFIIS and Pol II interaction? Are there any major conformational changes between the unbound state and the bound state of TFIIS? If yes, what are the driving forces responsible for these conformational changes of TFIIS? Here we employ the molecular dynamics (MD) simulation method, which allows us to study Pol II and TFIIS at the atomistic scale. In this investigation, we focus on two molecular aspects of TFIIS: (1) the conformation of TFIIS in the Pol II-free (unbound) and Pol II-bound states and (2) the thermodynamics associated with the insertion of the catalytic domain III of TFIIS into Pol II after an initial binding to Pol II. Since Pol II is a very large protein complex consisting of 12 subunits [13] and has more than 4000 amino acid residues, we consider a combination of all-atom and coarse-grained models. To the best of our knowledge, this study is the first attempt to simulate the dynamics of the Pol II-TFIIS complex.

## Materials and Methods

To study the conformations of TFIIS and related thermodynamics properties, we employed two systems: (1) Pol II-bound and (2) Pol II-free TFIIS systems. For each system, we prepared both all-atom (AA) and coarse-grained (CG) models, and conducted MD simulations to obtain the equilibrium structures and to calculate their physical quantities. Next, we used the umbrella sampling method with the CG models to perform free energy calculations to understand the thermodynamics associated with the conformational change of TFIIS upon binding to and dissociating from Pol II.

### All-atom simulations

The Pol II-TFIIS complex crystal structure (PDB ID: 3PO3 [12]) was adapted in our simulation to build our initial atomistic models. The complex contains a RNA polymerase II (12 subunits), a TFIIS, a RNA transcript, DNA template and non-template fragments, and metal ions. Note that in this PDB structure, the domain I of TFIIS (a N-terminal four-helix bundle) is omitted. It is well documented that the domain I is not essential in the Pol II binding and transcript cleavage and reactivation process [4], [11], [14] and is only involved in transcription initiation [15], [16]. Additionally, to stop transcript cleavage process during protein crystallization, a site-specific mutation of two catalytic residues (D290A/E291A) was introduced in TFIIS. This mutation is located at the a loop tip of domain III and is unlikely to change overall TFIIS structure and its binding with Pol II [12]. Since our main focus is on understanding the overall conformational change of TFIIS upon Pol II binding and insertion of TFIIS prior to the cleavage reaction rather than the cleavage reaction itself, we employed the same mutant as our primary TFIIS protein for the Pol II-TFIIS complex.

Based on the PDB structure, we prepared the initial structure of Pol II-TFIIS complex for simulation. The missing residues in the surface loops were filled using SWISS-MODEL [17], a protein structure homology-modeling server (http://swissmodel.expasy.org), as well as another Pol II structure at the elongation stage [18]. From this preprocessing, we obtained the necessary coordinates of Pol II and TFIIS with associated metal ions. To simplify the model, the RNA and DNA segments were removed because they are located far away from Pol II-TFIIS interface and unlikely to be influential on the overall conformation of TFIIS. The backtracked RNA fragment is interacting only with a small portion of the catalytic domain. Position-restraints on the Pol II were applied to maintain Pol II transcribing complex geometry during the simulation. To parameterize the bonded and non-bonded interactions between atoms, we used AMBER ff99SB force field [19] with ZAFF (Zinc Amber Force Field) [20] for the zinc ribbon motif in the domain III. TIP3P [21] water molecules and Na^+^ counterions were added to the system to achieve an electrically neutral solvate system (see Table 1 for details). Energy minimization was performed and then the system was slowly heated up from 0 K to 300 K prior to the NPT production run. NPT simulation was carried out at T = 300 K at 1 bar. To be consistent with the in vitro experiments performed at room temperature, we chose 300 K for our simulations. Temperature and pressure were regulated by Langenvin dynamics and the AMBER algorithm [22] based on Berendsen weak coupling [23], respectively. The particle mesh Ewald method [24] with a cutoff length of 10 Å was employed for electrostatic interactions, and the same cutoff length was also applied for the van der Waals interactions. The total NPT simulation time was 100 ns and the coordinates of the system were saved every 5 ps during the simulation.

**Table 1:** AA and CG Model Systems.

	TFIIS (164 residues)	TFIIS (164 residues)+Pol II (4014 residues)
**AA** Model	TFIIS+1 Zn+7 Cl^−^+46325 H_2_O (total: 141579 atoms)	Pol II+TFIIS+8 Zn+1 Mg+61 Na^+^+156178 H_2_O (total: 536427 atoms)
**CG** Model	TFIIS+1 Zn+7 Cl^−^+31150 4H_2_O (total: 31521 beads)	Pol II+TFIIS+8 Zn+1 Mg+61 Na^+^+52990 4H_2_O (total: 62485 beads)

The structure of TFIIS in the Pol II-free state is not available. Thus, the coordinates of TFIIS with a zinc ion were extracted from the Pol II-TFIIS complex (PDB ID: 3PO3) as the initial TFIIS structure for the MD simulation of the unbound TFIIS. The equilibrium structures of TFIIS in the Pol II-free state can be found by a long-time-scale molecular dynamic simulation. TFIIS was then solvated with TIP3P water and neutralized by adding Cl^−^ counterions to the system (see Table 1). The same force field and simulation parameters and procedures were used for the simulations of the unbound TFIIS and Pol II-bound TFIIS complex (described above). The total NPT simulation time was also 100 ns, and we saved the coordinates at 1 ps intervals. In this case, in addition to the mutant TFIIS simulation, we also carried out nine 60 ns simulations of a wild-type TFIIS with the same protocol used in the mutant case to explore the conformational space of the wild-type TFIIS. All AA simulations were conducted using the AMBER simulation package [22]. The protonation states of residues in the AA models were assigned using the PROPKA program [25] and the H++ server [26] (http://biophysics.cs.vt.edu/index.php).

### Coarse-grained simulations

To explore the conformational space of TFIIS extensively, we also performed CG MD simulations. The CG model we used is the MARTINI model [27], [28], developed by the Marrink group. The basic principle of MARTINI coarse-graining is that four heavy atoms are mapped into a single particle. Using the script (martinize.py) available from the Marrink group website (http://md.chem.rug.nl/cgmartini), we were able to convert the atomistic structures to the corresponding coarse-grained structures. In this conversion, we preserved the protonation states of residues to be the same as those used in the AA model. Since some AMBER residues were not defined in MARTINI force field, we created force fields for the missing residues by modifying the force field parameters from similar residues. For example, we made a CG force field for the AMBER residue HIP (the protonated form of histidine (HIS)) by adding charge to the HIS residue and changing the bead type to charged one. In order to implement the zinc ribbon motif in the CG model, we used the ZAFF parameters with distance-restraint forces to maintain the motif structure.

In this coarse-graining description, the total number of particles is significantly reduced compared to the atomistic models, allowing us to perform long simulations and computationally expensive free energy calculations. The details of the system composition for each case are shown in Table 1. We carried out CG simulations using GROMACS 4.5 [29] and we used the tools in the GROMACS package to analyze the simulation data. The same simulation protocol was used for both AA and CG simulations: energy-minimization, heating from 0 K to 300 K and then running the NPT simulation at 300 K at 1bar pressure. The pressure and temperature algorithms were from the Berendsen coupling scheme [23]. The electrostatic interactions were calculated using the shift method defined in GROMACS with a cutoff length of 12 Å, and this cutoff length was also used for the van der Waals interactions. The trajectories were saved every 200 ps and the total NPT simulation time was 6 µs for each case.

### Free energy calculations

To study thermodynamics associated with the conformational changes of TFIIS upon binding to Pol II, we performed free energy calculations using the CG model with the umbrella sampling technique [30]. Specifically, we calculated the potential of mean force (PMF), a free energy change as a function of reaction coordinate, by employing the weighted histogram analysis method (WHAM) [31]. The system setup and calculation procedures are described in detail in Results and Discussion.

## Results and Discussion

### TFIIS has two distinct conformation states: open and closed forms

**Figure 2:**
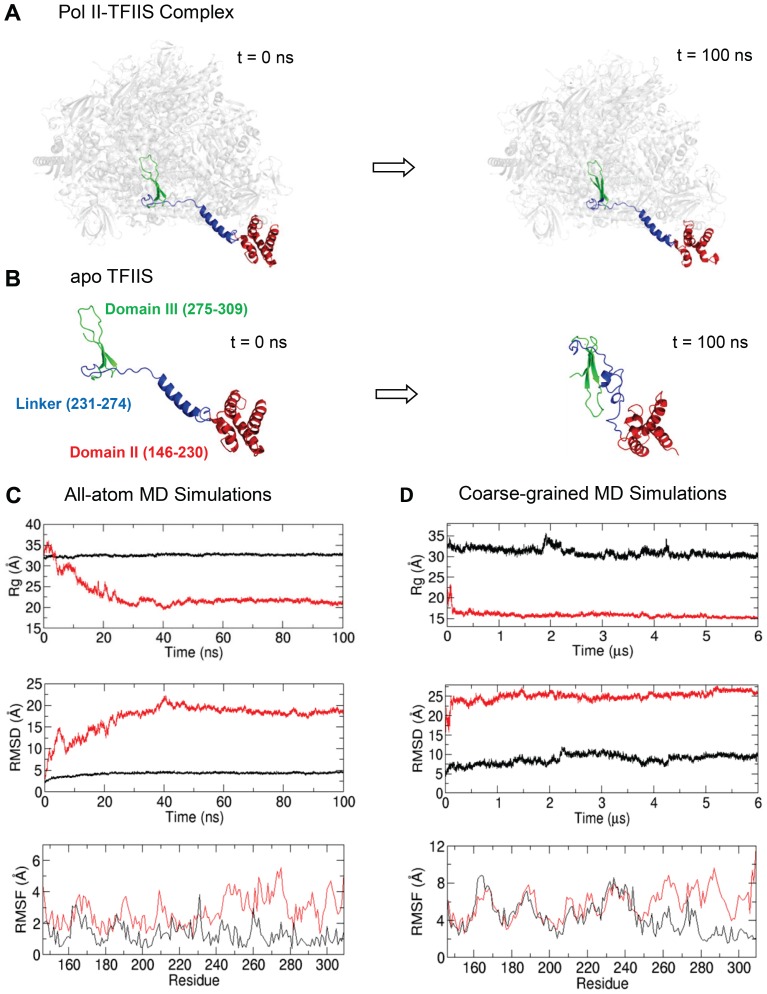
MD simulation results of Pol II-TFIIS complex and apo TFIIS. The initial (left) and final (right) structures of Pol II-TFIIS complex (A) and apo TFIIS (B) from all-atom MD simulations. MD simulation results for all-atom models (C) and coarse-grained model (D). In (C) and (D), the black and red curves represent the cases of the Pol II-TFIIS complex and apo TFIIS, respectively.

In order to understand the dynamics and equilibrium structures of TFIIS in the Pol II-bound and Pol II-free states, we performed 100 ns MD simulations starting from the initial structures shown in the left panels of Figures 2A and 2B. Intriguingly, we observed two distinct stable conformations of TFIIS: extended (open) and collapsed (closed) forms (right panel of Figures 2A and 2B). In the Pol II-bound state, TFIIS stays in an extended conformation (open form) with its catalytic domain III inserted into the secondary channel of Pol II throughout the simulation. In sharp contrast, the simulation of TFIIS in the absence of Pol II reveals a significant conformational change that TFIIS switches to a collapsed conformation (closed form) within a relatively short time period and remains throughout the simulation, suggesting that this extended conformation is unstable for TFIIS in the unbound state. As shown in the top and middle panels of Figure 2C, there are significantly changes of the root-mean-square deviation (RMSD) and the radius of gyration (Rg) of TFIIS in the absence of Pol II (red) during the simulation, indicating global conformational changes from the open form to the closed form, whereas there are almost no changes of RMSD and RG in the simulation of the Pol II-bound TFIIS (black).

Since the TFIIS reached equilibrium after ∼60 ns (top and middle panels in Figure 2C), we calculated the root-mean-square fluctuation (RMSF) per residue over the last 40 ns trajectory (60–100 ns) (bottom panel in Figure 2C). The RMSF of the linker (residues 231–274) and the domain III (residues 275–309) significantly decreases in the Pol II-bound state (black) in comparison with the Pol II-free state (red). The result also indicates that when the geometric confinement by Pol II is removed, the linker region and the domain III are more flexible, which may be responsible for the large-scale conformational change observed in the simulation. In fact, the additional RMSD analysis for each domain shows that the RMSD change in the linker region is the largest in the overall conformational change, i.e. Domain II: ∼4 Å, Linker: ∼12 Å, and Domain III: ∼3 Å.

We further studied the secondary structural change of TFIIS. Interestingly, the secondary structures of domains II and III are conserved during the MD simulations of both Pol II-free and Pol II-bound states. In the presence of Pol II, the secondary structure of the linker region is also relatively well-preserved (Figure S1A) but it changes dynamically in the absence of Pol II (Figure S1B). Additionally, we attribute the large RMSF peak around residue 230 in the presence of Pol II (Figure 2C) to the dynamical change of secondary structure shown in Figure S1A.

In addition to the Pol II-free mutant TFIIS simulation, we also carried out nine independent Pol II-free 60 ns MD simulations of wild-type TFIIS from the same initial structure. Consistent with previous simulation with the mutant TFIIS, we observed similar stable closed forms of TFIIS in the equilibrated structures of all nine independent simulations (see the Rg analysis in Figure S2). The exact conformations of these closed forms from the ten simulations (1 mutant+9 WT-type) are somewhat different, suggesting that the TFIIS might adopt multiple closed forms in the absence of Pol II, which is mainly due to the highly flexible linker region. Since TFIIS only has a biological role when it is bound to Pol II, it might not be necessary to have one dominant closed form, as long as all of the closed forms change to the open form upon binding to Pol II. Additionally, the flexibility of the linker may be necessary to easily insert and withdraw the catalytic domain from the active site of Pol II during the reactivation process.

To sufficiently explore the whole conformational space of TFIIS, we further extended our simulation in a much longer time scale (µs) using CG MD simulation. From the simulation, we also calculated Rg, RMSD, and RMSF, as we did in the AA simulations. For the Rg calculation, we took a trajectory from 3 µs to 6 µs. The results of CG MD simulations are consistent with those of the AA simulations. The TFIIS maintains the open form in the Pol II-bound state (Figure S3A), whereas TFIIS rapidly collapses and keeps in the closed form in the absence of Pol II (Figure S3B). The Rg, RMSD and RMSF plots reveal a similar pattern as those of AA simulation, when the systems reach equilibrium (see Figure 2D). Because of different time scales (ns vs. µs) in the plots, the equilibration may look different in the AA and CG cases, but when we plot Rg and RMSF for the initial 100 ns time interval, we see that they have similar equilibration time and time-transient behavior (see Figure S4). Taken together, both the CG (6-µs) and AA (100-ns) simulation results reach the same conclusion of TFIIS conformation: the open form in the presence of Pol II and the closed form in the absence of Pol II.

Notably, our CG and AA simulation results showed good agreement, where they captured similar conformations of TFIIS. This consistency allowed us to study TFIIS further using the CG model. Specifically, the relatively low computational cost of the CG model enabled us to study the thermodynamics associated with the large-scale conformational change, which would otherwise require enormous computational resources and time if the AA model were used.

### Conformational change of TFIIS in the absence and the presence of Pol II

Our simulation results of two distinct conformations of TFIIS raised a few fundamental questions: 1) why does the conformation of TFIIS switch from the open form to the closed form in the absence of Pol II and why does it stay in the open form in the presence of Pol II? In other words, what are the free energy differences between the two TFIIS conformation forms in the presence and absence of Pol II? 2) what are the driving forces for the conformational changes? These questions are important to understand the functional interplay between Pol II and TFIIS.

To investigate the aforementioned questions quantitatively, we calculated the free energy difference between the two conformation forms in the presence and absence of Pol II. Specifically, we calculated the potential of mean force (PMF). Since these two conformations are well-characterized by the inter-domain distance, we used the distance between two centers of mass of domains II and III as the reaction coordinate in the PMF calculation.

**Figure 3:**
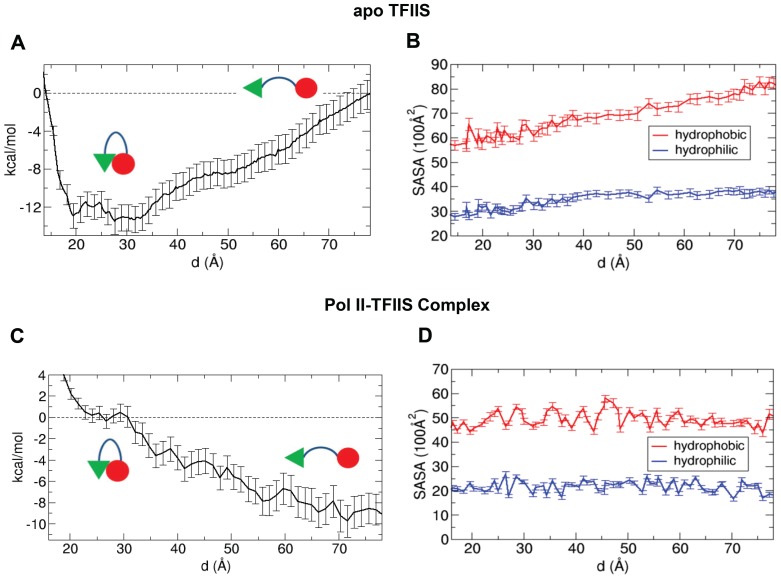
PMF and SASA of apo TFIIS and Pol II-TFIIS complex. (A) PMF of coarse-grained TFIIS as a function of the distance between the domains II and III, and (B) hydrophobic and hydrophilic solvent accessible surface area (SASA) of coarse-grained TFIIS in the absence of Pol II. Note that, at small distances (<∼20 Å), the PMF increases while the hydrophobic SASA decreases. The increase in the PMF is due to the steric repulsion between the two domains. The value of PMF at the largest distance (∼78 Å) is set to be zero. (C) PMF and (D) SASA in the presence of Pol II. The value of PMF at the metastable states (d = ∼25 Å) is set to be zero.

**A. TFIIS favors the closed form in the Pol II-free state.** For the conformational change of TFIIS observed in the absence of Pol II, we calculated the free energy difference using the CG model with the umbrella sampling technique. For the calculation, we prepared 47 windows from a pulling simulation, which represent the inter-domain distances ranging from 11 Å to 78 Å, and then we ran a 50 ns MD simulation for each window. Here, the inter-domain distance is a good reaction coordinate for describing the state of TFIIS in that the inter-domain distance is well defined and also well correlated with the radius of gyration (see Figure S5). For the pulling simulation, we applied force to bring two domains together from the extended form of TFIIS, and in this way, we prepared the initial structures for the umbrella sampling including the closed forms. By taking the last 40 ns trajectory in each window, we calculated the PMF by solving the WHAM (weighted histogram analysis method) equation. The result is displayed in Figure 3A. From the plot, it is clear that the closed form is thermodynamically more favorable than the open form in the absence of Pol II. The free energy difference is about 13 kcal/mol, which is much larger than thermal energy at 300 K, ∼0.6 kcal/mol. Therefore, it is likely that in solution, the dominant form of unbound TFIIS is the closed form (open form: closed form = 1∶∼e^(13/0.6)^≈1∶10^9^).

Since the conformational change may be associated with interactions with water, we calculated the hydrophobic and hydrophilic areas accessible for water (solvent accessible surface area, SASA) in the sampled states. For this analysis, we used the GROMACS analysis tool (g_sas), and the result is shown in Figure 3B. As the inter-domain distance decreases, the hydrophobic area decreases while the hydrophilic area does not change much. This decreasing of hydrophobic area is correlated with the change in the PMF up to a distance of ∼30 Å. This result suggests that hydrophobic interaction is the driving force for the conformational change of TFIIS from the open form to the closed form.

**B. TFIIS favors the open form in the Pol II-bound state.** To investigate the thermodynamic details of the TFIIS conformation change during TFIIS recruitment and insertion into the Pol II active site, we calculated the free energy difference between the inserted (open) and the non-inserted (closed) state of TFIIS in the Pol II-bound state. Here we focused on the insertion of domain III assuming that the partial binding of domain II to Pol II is already established. The binding position of domain II with respect to Pol II was determined from the PDB structure of Pol II-TFIIS complex. To make the free energy calculation feasible, we again used the CG model. As we mentioned in Materials and Methods, we applied position-restrained potential to the Pol II to maintain its structure without RNA/DNA chains, but to give some flexibility during the insertion, we allowed ∼14% of the total residues (1301 out of 9070 beads) which are near the insertion pathway, to move freely. Additionally, to introduce the partial binding between TFIIS and Pol II, we also applied a position-restrained potential to the domain II of TFIIS.

For the PMF calculation, we again used the umbrella sampling and prepared the initial structures for the sampling windows by performing a pulling simulation. Specifically, from a bound state, we pulled out the TFIIS domain III from the active site of Pol II and brought it to the domain II at a fixed position. Thus, the reaction coordinate is again the distance between the two domains. In this study we considered only one pathway which was obtained from the direct pulling simulation and this pathway is the shortest path in bringing two domains together under the given constraint to the domain II. However, in reality, there could be many other possible pathways during the insertion of domain III. For the umbrella sampling, we prepared 67 windows along the reaction coordinate with the distances ranging from 12 Å to 78 Å, and we ran two consecutive MD simulations at 450 K (20 ns) and 300 K (40 ns) for each window. The simulation at 450 K was performed for relaxing the system obtained from the pulling simulation and allowing the system to easily find global free-energy minima. The last 18 ns trajectory was used for the PMF calculation and the result is depicted in Figure 3C. As shown in the equilibrium MD simulation, the open form is much stable than the closed form. The free energy difference between the two conformations is about 9 kcal/mol. Therefore, in this CG model, the equilibrium is biased toward the open form and it is very unlikely to observe the closed form when the TFIIS is interacting with Pol II (open form: closed form = ∼e^(9/0.6)^≈10^6^∶1). This conclusion implies that once TFIIS partially binds to Pol II, the catalytic domain III can be inserted into the active site of Pol II along the path we obtained during the pulling simulation.

As we did in the absence of Pol II, we also performed the SASA analysis in the presence of Pol II and the result is shown in Figure 3D. In contrast to the Pol II-absent case, the solvent-accessible surface areas do not change significantly as the inter-domain distance decreases. This implies that the hydrophobic and the hydrophilic interactions with water do not contribute significantly to the conformational change of TFIIS. Instead, the conformational change is mainly driven by protein-protein interactions between TFIIS and Pol II.

## Conclusions

**Figure 4:**
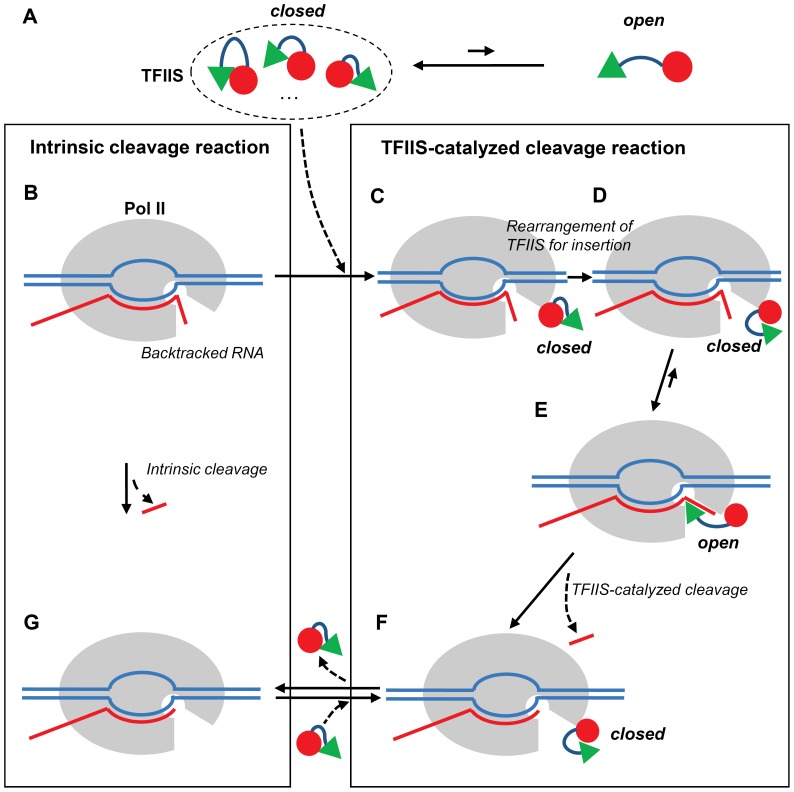
Schematic diagram of the reactivation process from the arrested state of Pol II. (A) TFIIS in solution. (B) Backtracked state of Pol II. (C) State of Pol II with an initial partially-bound TFIIS. (D) State of Pol II with a partially-bound TFIIS for the insertion of catalytic domain. (E) State of Pol II with a fully-bound TFIIS. (F) Elongation state of Pol II with a partially-bound TFIIS. (G) Normal elongation state of Pol II.

In this work, we explored the conformational space of TFIIS with Pol II using AA and CG MD simulations to better understand the interaction between Pol II and TFIIS as well as reactivation process of Pol II by TFIIS. From the simulations, we obtained two major conformations of TFIIS, the closed and the open forms. When TFIIS is in aqueous solution (unbound state), it prefers the closed form in order to minimize the hydrophobic surface area. According to the PMF calculation, this closed form is so stable that it is unlikely to switch to the open form, as depicted in Figure 4A. However, when TFIIS is fully bound to Pol II, TFIIS has the stable open form until catalytic reaction occurs, as observed in the X-ray crystal structure. This open form represents the active state of TFIIS where the catalytic domain is inserted into the active site of Pol II. The stability of the open form is confirmed by a large free energy difference in the PMF calculation, which shows the free energy of open form is much lower than the one of closed form. Taken together, these results indicate that once the closed TFIIS in solution binds to Pol II (see Figures 4C and 4D), TFIIS undergoes a major conformational change to the open form with the insertion of the catalytic domain of TFIIS (see Figure 4E).

This computational study gives us some novel insights into the reactivation process of Pol II by TFIIS. Here we summarize a general mechanism of TFIIS binding, transcript cleavage, and reactivation process in Figure 4. The left panel describes the intrinsic cleavage reaction in which Pol II itself cleaves the backtracked RNA without the help of TFIIS, while the right panel describes the TFIIS-catalyzed cleavage reaction. The TFIIS-catalyzed reaction is a much faster reaction than the intrinsic cleavage reaction, and it is initiated by the binding between TFIIS and Pol II. Probably, TFIIS could have multiple closed conformations in the unbound state (Figure 4A) but once it binds to Pol II through the domain II (Figure 4C), its overall conformation is adjusted on the Pol II surface and changed to the correct closed conformation for the insertion of catalytic domain into Pol II (Figure 4D). Then the catalytic domain III is inserted into the active site of Pol II and the TFIIS-catalyzed cleavage reaction takes place (Figure 4E). During this process, the conformational change of TFIIS occurs from the closed form to the open form. After the cleavage reaction is completed, the TFIIS domain III is removed from Pol II active site for the entrance of NTP (Nucleoside triphosphate), a building block of RNA, in the new round of RNA transcript synthesis (Fig. 4F). Noticeably, this model indicates that TFIIS can be still bound to Pol II even after the expulsion of its catalytic domain III from Pol II active site during Pol II transcription reactivation. This scenario may suggest multiple rounds of backtracking and transcript cleavage per TFIIS association/dissociation cycle. In the case of complete dissociation of TFIIS, the state of Pol II goes to the normal elongation state in Figure 4G and TFIIS has the closed form depicted in Figure 4A.

Our study also paves the way for future studies. To completely understand the reactivation process by TFIIS, the next step in the direction would be to investigate the catalytic reaction occurring inside of Pol II and the removal process of the TFIIS catalytic domain III from the Pol II active site (Figures 4E and 4F). To this end, a detailed atomistic simulation of the chemical reaction that may include the full modeling of backtracked RNA transcript and metal ions with a wild-type TFIIS will be performed, and how the transcript cleavage reaction is coupled with TFIIS domain III release will be studied.

## Supporting Information

Figure S1 **Secondary structure analysis for TFIIS in the AA MD simulations.** (A) TFIIS in complex with Pol II. (B) TFIIS in the absence of Pol II.
(TIFF)

Figure S2 **Time evolutions of radius of gyration (Rg) of TFIIS in nine wild-type TFIIS MD simulations.**
(TIFF)

Figure S3 **Coarse-grained** **MD** **simulation results of Pol II-TFIIS complex and apo TFIIS.** (A) Initial (left) and final (right) structures of Pol II-TFIIS complex from the coarse-grained simulation. (B) Initial (left) and final (right) structures of apo TFIIS from the coarse-grained simulation.
(TIFF)

Figure S4 **Comparison of the all-atom and coarse-grained MD simulations for the initial 100 ns.** Radius of gyration (Rg) (A) and root mean square displacement (RMSD) (B) of apo TFIIS from the all-atom (AA) and coarse-grained (CG) MD simulations.
(TIFF)

Figure S5 **Radius of gyration and the domain-domain distance in the coarse-grained MD simulations.** (A) Radius of gyration (Rg) and the domain-domain distance of the apo TFIIS from the coarse-grained (CG) MD simulation. The domain-domain distance is defined by the distance between the centers of mass of domain II and domain III. The inset is the results during the first 0.2 µs. (B) Rg and the domain-domain distance of Pol II-TFIIS complex from the CG MD simulation.
(TIFF)
