# Supplementary figures and images for: Molecular Dynamics Simulation Study of Conformational Changes of Transcription Factor TFIIS during RNA Polymerase II Transcriptional Arrest and Reactivation
Source: PLoS One. 2014 May 19;9(5):e97975. doi: 10.1371/journal.pone.0097975 (PMC4026522; doi:10.1371/journal.pone.0097975)

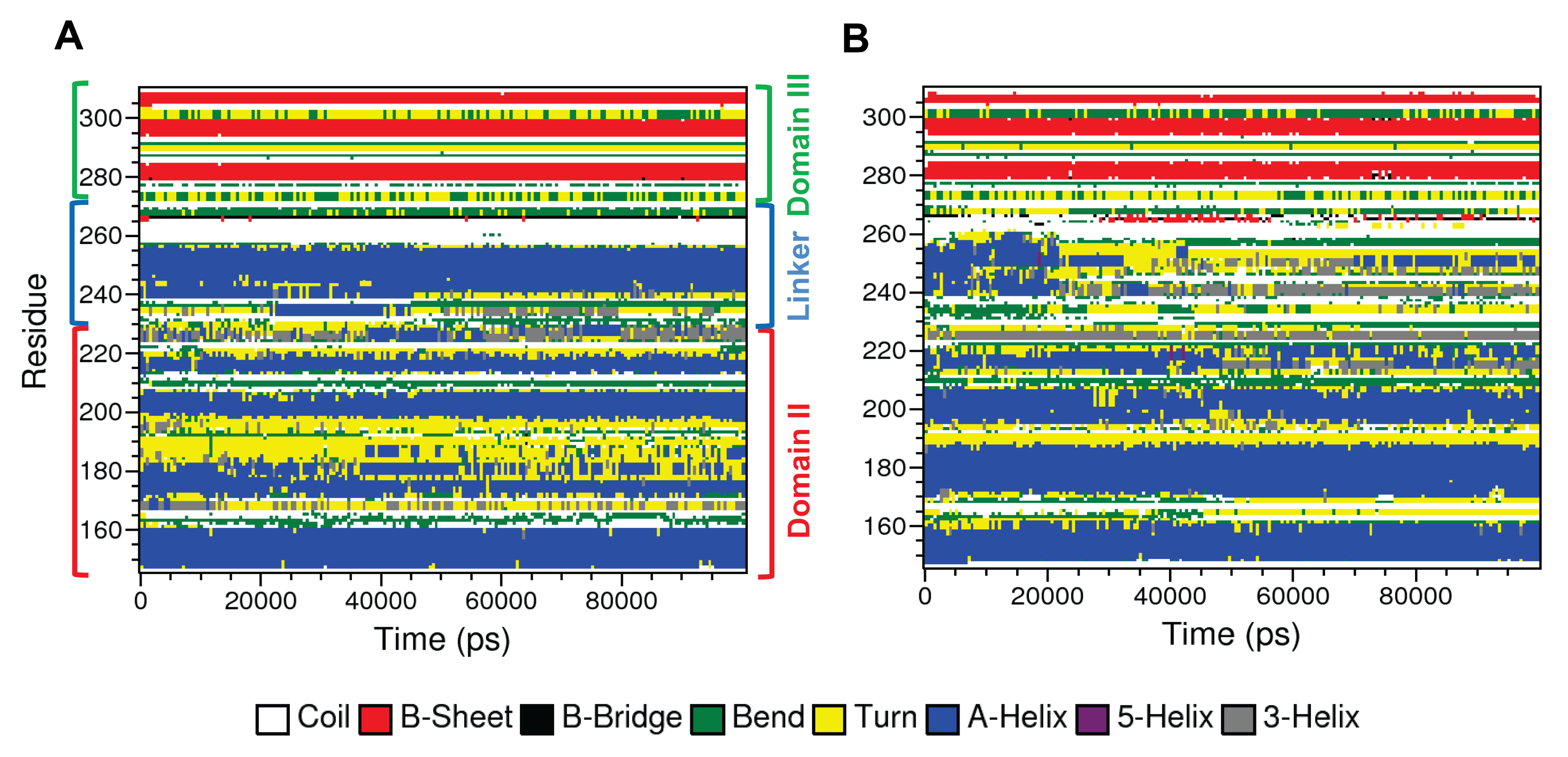

Supplement: Figure S1 — Secondary structure analysis for TFIIS in the AA MD simulations. (A) TFIIS in complex with Pol II. (B) TFIIS in the absence of Pol II. (TIFF) [file pone.0097975.s001.tiff]

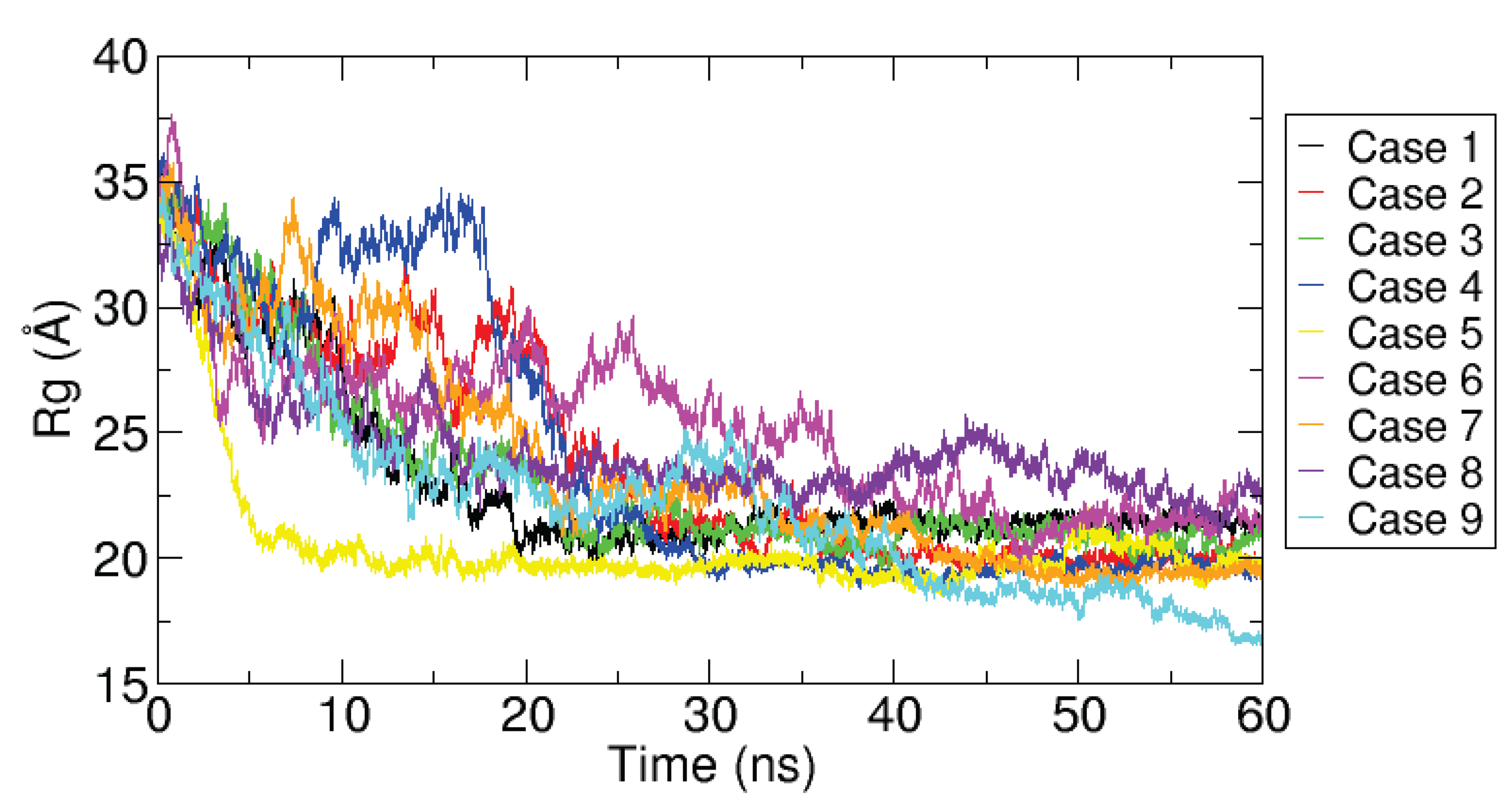

Supplement: Figure S2 — Time evolutions of radius of gyration (Rg) of TFIIS in nine wild-type TFIIS MD simulations. (TIFF) [file pone.0097975.s002.tiff]

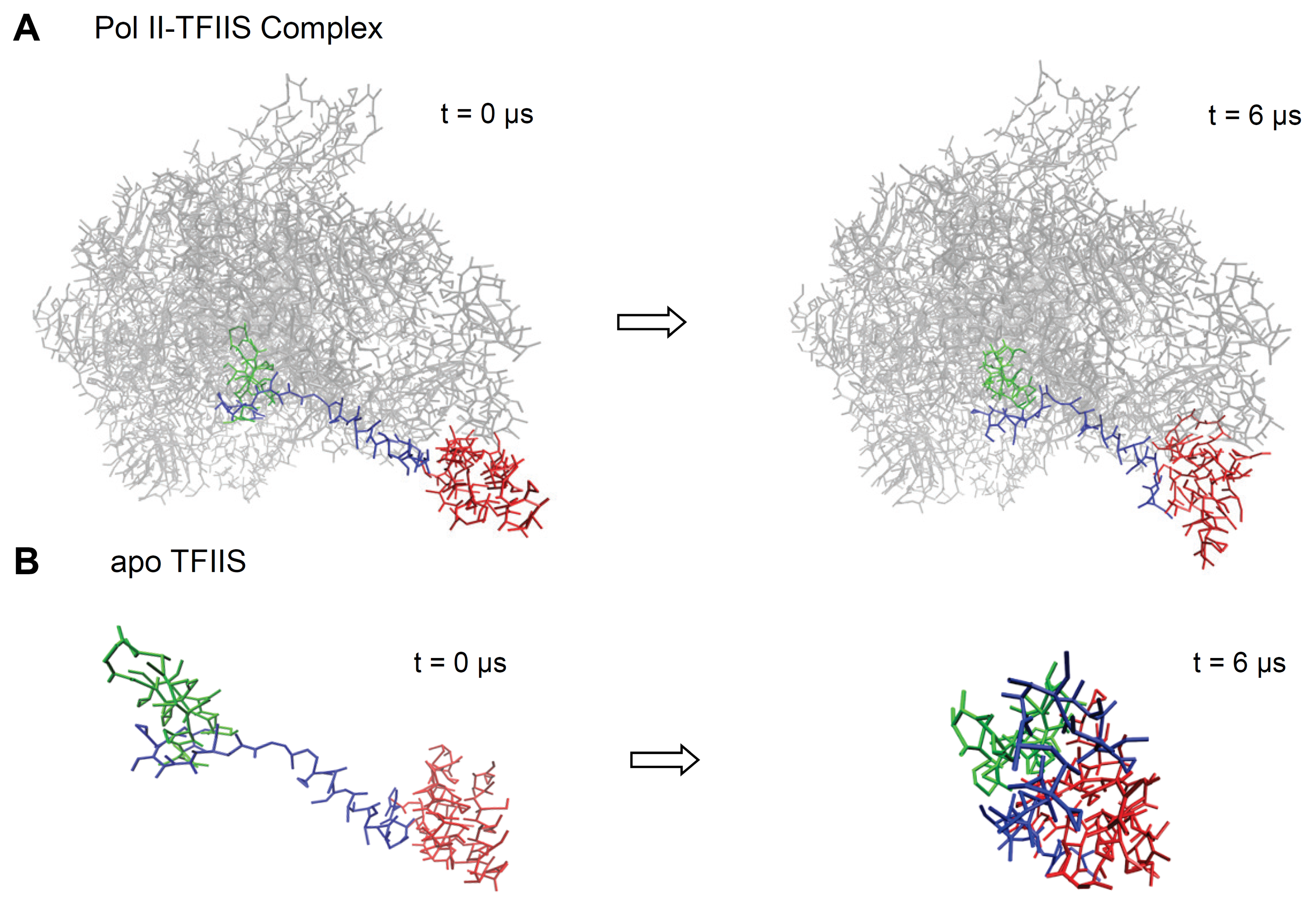

Supplement: Figure S3 — Coarse-grained MD simulation results of Pol II-TFIIS complex and apo TFIIS. (A) Initial (left) and final (right) structures of Pol II-TFIIS complex from the coarse-grained simulation. (B) Initial (left) and final (right) structures of apo TFIIS from the coarse-grained simulation. (TIFF) [file pone.0097975.s003.tiff]

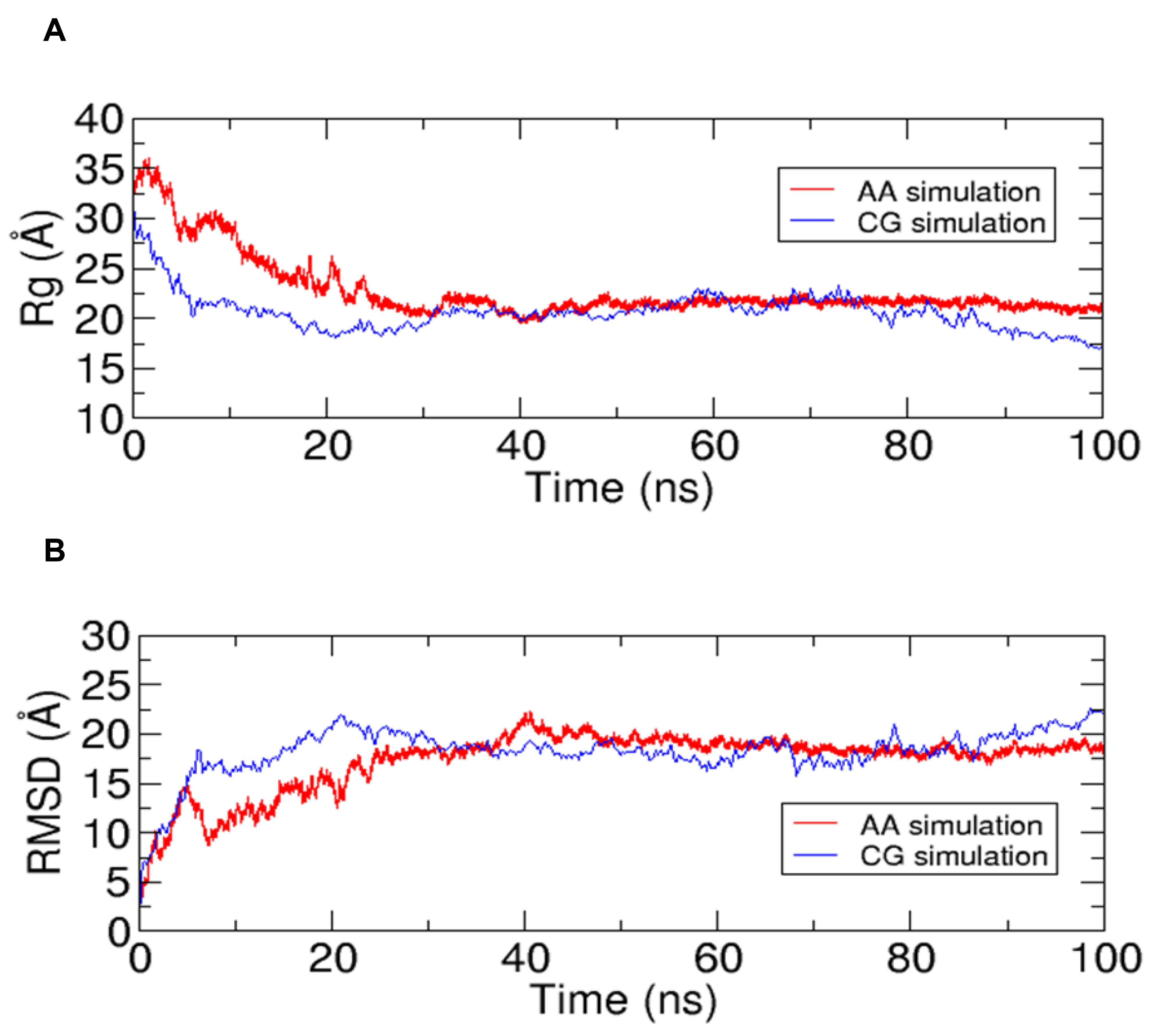

Supplement: Figure S4 — Comparison of the all-atom and coarse-grained MD simulations for the initial 100 ns. Radius of gyration (Rg) (A) and root mean square displacement (RMSD) (B) of apo TFIIS from the all-atom (AA) and coarse-grained (CG) MD simulations. (TIFF) [file pone.0097975.s004.tiff]

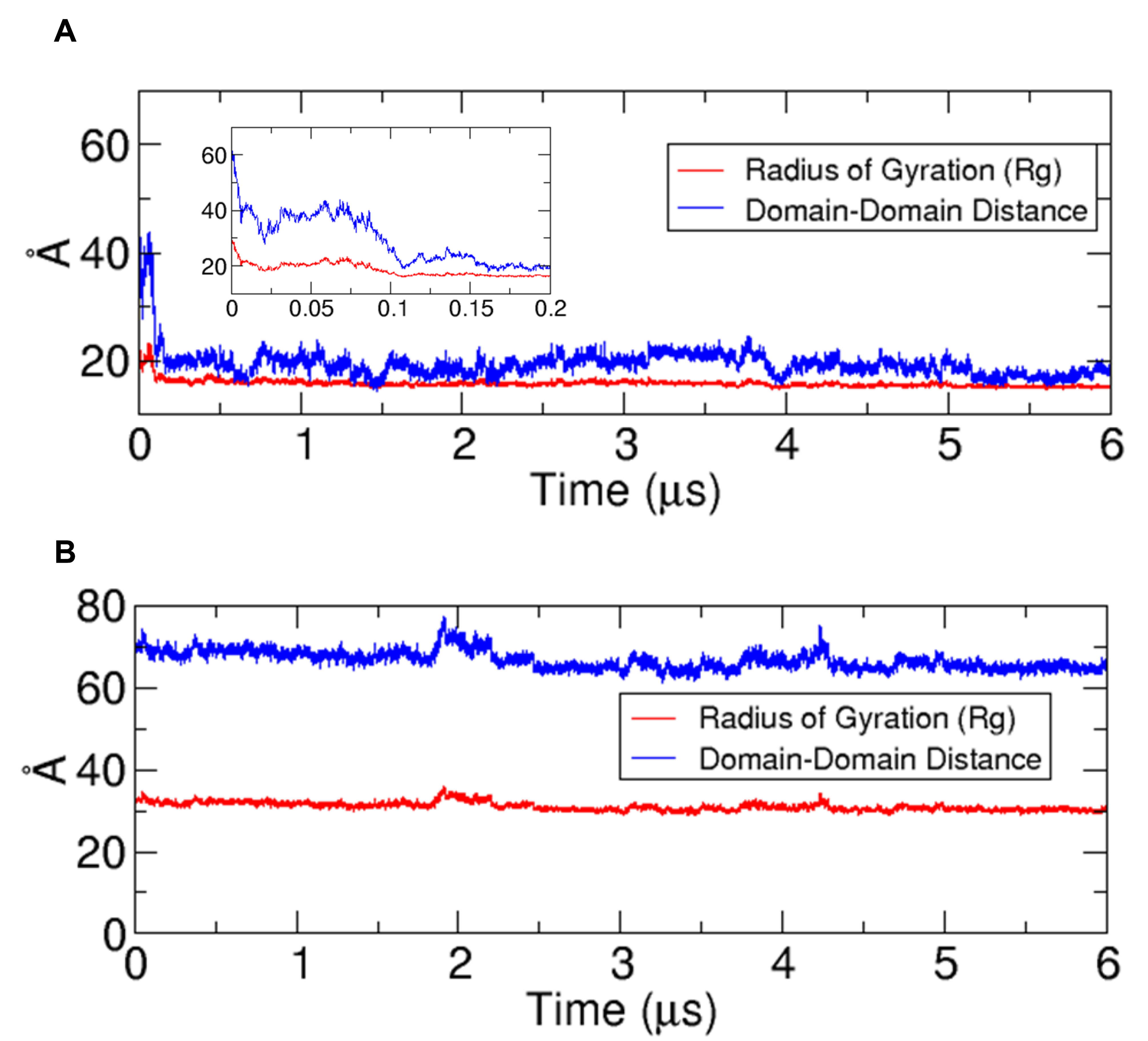

Supplement: Figure S5 — Radius of gyration and the domain-domain distance in the coarse-grained MD simulations. (A) Radius of gyration (Rg) and the domain-domain distance of the apo TFIIS from the coarse-grained (CG) MD simulation. The domain-domain distance is defined by the distance between the centers of mass of domain II and domain III. The inset is the results during the first 0.2 µs. (B) Rg and the domain-domain distance of Pol II-TFIIS complex from the CG MD simulation. (TIFF) [file pone.0097975.s005.tiff]
